# Supplementary figures and images for: Untargeted metabolomics confirms the association between plasma branched chain amino acids and residual feed intake in beef heifers
Source: PLoS One. 2022 Nov 29;17(11):e0277458. doi: 10.1371/journal.pone.0277458 (PMC9707789; doi:10.1371/journal.pone.0277458)

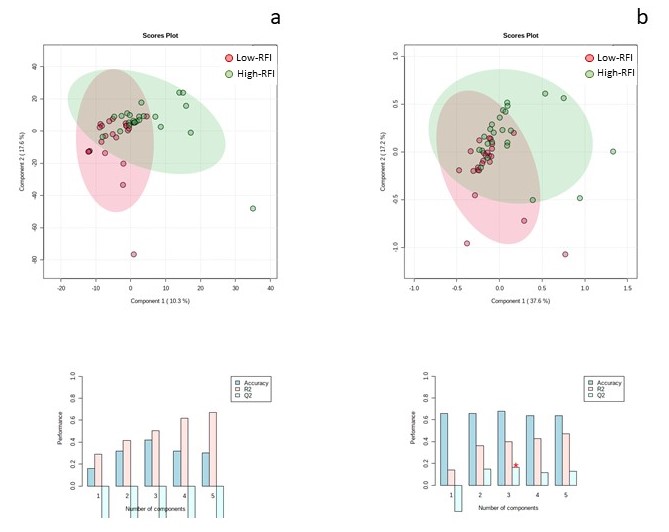

Supplement: S1 Fig — Partial least square discriminant analysis (PLS-DA) for residual feed intake (RFI) on the basis of: a) total metabolomic dataset (3,457 ions) or b) only confirmed metabolites (annotation class 1). Score plot, and cross validation values are presented in the top and bottom of each figure panel, respectively. Red and green dots depict Low- and High-RFI, respectively. (JPG) [file pone.0277458.s001.jpg]
